# Dosimetry Analysis in Non-brain Tissues During TMS Exposure of Broca’s and M1 Areas

**DOI:** 10.3389/fnins.2021.644951

**Published:** 2021-02-19

**Authors:** Jose Gomez-Tames, Keisuke Tani, Kazuya Hayashi, Satoshi Tanaka, Shoogo Ueno, Akimasa Hirata

**Affiliations:** ^1^Department of Electrical and Mechanical Engineering, Nagoya Institute of Technology, Nagoya, Japan; ^2^Center of Biomedical Physics and Information Technology, Nagoya Institute of Technology, Nagoya, Japan; ^3^Hamamatsu University School of Medicine, Hamamatsu, Japan; ^4^Department of Biomedical Engineering, Graduate School of Medicine, The University of Tokyo, Tokyo, Japan

**Keywords:** perception threshold, pain threshold, dosimetry, nerve model, TMS, standardization, side-effects

## Abstract

For human protection, the internal electric field is used as a dosimetric quantity for electromagnetic fields lower than 5–10 MHz. According to international standards, in this frequency range, electrostimulation is the main adverse effect against which protection is needed. One of the topics to be investigated is the quantification of the internal electric field threshold levels of perception and pain. Pain has been reported as a side effect during transcranial magnetic stimulation (TMS), especially during stimulation of the Broca’s (speech) area of the brain. In this study, we designed an experiment to conduct a dosimetry analysis to quantify the internal electric field corresponding to perception and pain thresholds when targeting the Broca’s and M1 areas from magnetic stimulator exposure. Dosimetry analysis was conducted using a multi-scale analysis in an individualized head model to investigate electrostimulation in an axonal model. The main finding is that the stimulation on the primary motor cortex has higher perception and pain thresholds when compared to Broca’s area. Also, TMS-induced electric field applied to Broca’s area exhibited dependence on the coil orientation at lower electric field threshold which was found to be related to the location and thickness of pain fibers. The derived dosimetry quantities provide a scientific rationale for the development of human protection guidelines and the estimation of possible side effects of magnetic stimulation in clinical applications.

## Introduction

There has been concern about human safety under exposure to electromagnetic fields. To protect humans from electromagnetic exposure, safety guidelines/standards have been developed by international standardization bodies (ICNIRP, 1998; International Commission on Non-Ionizing Radiation Protection, 2020; IEEE Std C95.1-2019, 2019). In the guidelines/standards for exposure to up to 100 kHz (5–10 MHz for brief pulse exposures), electrostimulation is the primary effect against which protection is needed. The IEEE ICES has published a research agenda for low-frequency exposure (Reilly and Hirata, 2016). One of the items for the peripheral nervous system is target tissue for electrostimulation.

One difficulty to conduct assessment of protection limits is that exposure levels from conventional appliances are well below the limit prescribed in the guidelines/standards and may not cause any stimulation at all. However, electrostimulation can occur during medical treatment; one example is transcranial magnetic stimulation (TMS), which is often used for the diagnosis of brain functions, neuro-rehabilitation, therapy for depression, and so on (Barker et al., 1985; Pascual-Leone et al., 1991; Pascual-Leone et al., 1993; Terao and Ugawa, 2002; Tanaka et al., 2011; Ziemann, 2011; Rossini et al., 2015).

During magnetic stimulation, pain has been reported as a side effect, especially during stimulation of the Broca’s (speech) area of the brain. Pain thresholds for both Broca’s area and M1 are different and significantly lower than the motor threshold as measured via motor evoked potentials (MEPs; Tani et al., 2020), which suggests that experiments might evoke pain sensation at the site of stimulation. This side effect generally becomes relevant when the intended target is in deep regions (Deng et al., 2014; Lerner et al., 2019; Gomez-Tames et al., 2020a). Safety and recommendations for TMS have been also published (Rossi et al., 2020).

In this study, we designed an experiment to explore the perception and pain thresholds when targeting the Broca’s and M1 areas for exposure to magnetic stimulators. The corresponding internal electric fields in the skin and muscles were then computed. In addition, multi-scale modeling, i.e., the combination of electromagnetics and nerve activation modeling, has been used to clarify the stimulation site on the tissue.

## Materials and Methods

### Participants

Twelve participants were recruited for each of the BA (21.2 ± 0.8 years, seven male and five female) and M1 (23.2 ± 3.1 years, nine male and three female). Four participants took part in both conditions. None of the participants had any contraindications to TMS, took any medication on a regular basis, or had a history of psychiatric or neurological diseases through questionnaires. Written informed consent was obtained from all participants before their participation. The study was approved by the ethical committee of the Hamamatsu University School of Medicine and was conducted in accordance with the Declaration of Helsinki.

### Magnetic Resonance Imaging

The head models of the 12 participants were constructed from T1- and T2-weighted images with acquisition parameters as follows: T1 MPRAGE sequence with TR/TE/FA/FOV/voxel size/slice number = 7.172 ms/2.12 ms/15°/256 mm/1.0 mm × 1.0 mm × 1.0 mm/196, and T2 with TR/TE/FOV/voxel size/slice number = 2502 ms/76.404 ms/256 mm/1.0 mm × 1.0 mm × 1.0 mm/196.

### Anatomical Head Model

**FIGURE 1:**
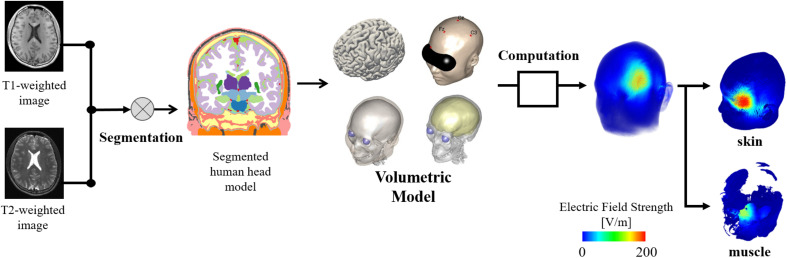
Individualized head models derived from MR images considering non-brain tissues. The model is used to obtain the induced electric field on skin and brain. In addition, a volumetric model of the head tissues is used as reference to determine the electric field values on different tissues.

The volume conductor of the head model was obtained by estimating the electrical conductivity values of the brain and non-brain tissues by applying deep learning based on MR images, as shown in Figure 1. The head tissues were segmented using in-house software equipped with the FreeSurfer brain imaging software package, as described previously (Laakso et al., 2015), and the FreeSurfer image analysis software (Fischl, 2012) was used to reconstruct the surfaces of the gray and white matter. Non-brain tissues were segmented from T1- and T2-weighted MRI using a semi-automatic procedure of region-growing and thresholding techniques.

### Measurement Protocol

**FIGURE 2:**
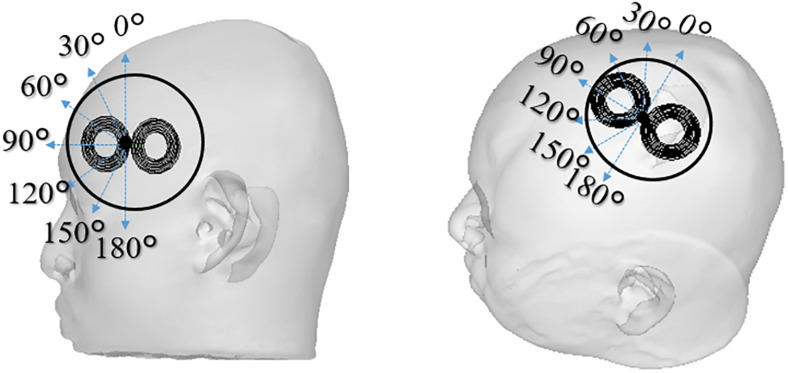
The TMS coil is placed in different coil orientations relative to the subject’s scalp used in the experiments (0°–180° with steps of 30°). Two scalp sites were investigated that corresponds to stimulation of Broca’s area and M1 of the left hemisphere. The reference coil orientations for 0° were defined as the inferior-superior orientation for the Broca’s area, and 45° inward relative to the anterior-posterior orientation for the M1. The orientation and positions are recorded to be used in the individualized dosimetry computation analysis.

The procedure to detect perception and pain thresholds was based on our previous study (Tani et al., 2020). In brief, all subjects were awake, sat comfortably in a reclining chair in a quiet environment, and were requested to relax. Single-pulse TMS was applied with a Magstim 200^2^ magnetic stimulator (Magstim Co., United Kingdom) connected to a double alpha BI coil (60 mm, Magstim Co., Ltd., United Kingdom). Perception/pain thresholds derived at two stimulation sites corresponding to stimulation of left Brodmann area 44 for Broca’area and the center of the hand knob (Yousry et al., 1997) area on the Brodmann area 4 for M1. The cortical areas were anatomically identified by the construction of a 3D cortical surface model of the individual participants using a frameless navigation system (Brainsight, Rogue Research Inc, Canada) based on the individual T1 image. A total of seven coil rotations were measured with approximately 5 min of rest between coil-orientation conditions for each target region. Figure 2 shows the definition of coil orientations over both target regions.

The subjects were instructed to report the presence or absence of scalp perception/pain after each stimulation. For scalp perception, the participants were instructed to report the presence or absence of a sensation of pressure or force. For the perception of pain, the participants were instructed to report the presence or absence of scalp-pain after each stimulation, regardless of the magnitude or type of pain. We used an adaptive staircase method (Tani et al., 2020) to determine the thresholds. The intensity decreased when perception/pain was reported and increased when perception/pain was not reported. A perception/pain threshold was defined as the minimum intensity that induced perception/pain in at least five of the ten trials. The thresholds are given as the percentage of the maximum stimulation output of the device (%MSO). This procedure was repeated for each coil orientation and the target area (M1 and Broca’s).

Each participant completed the experiment on two separate days. The perceptual/pain thresholds for the seven coil-orientation conditions were measured in a randomized order on the first day. The order of the coil-orientation conditions on the second day was set in reverse to the first day. For each coil-orientation condition, perceptual/pain thresholds were averaged from the two days to reduce the potential influence of sensory adaptation or fatigue. The total number of stimulations varied between 250 and 450 stimuli depending on the participants.

### Electromagnetic Computation

We assumed that the electric displacement current is negligible when compared to the conduction current (magneto-quasi-static approximation) and that the induced current does not perturb the external magnetic field (Plonsey and Heppner, 1967; Barchanski et al., 2005; Hirata et al., 2013). First, the electric scalar potential induced in the brain ϕ was determined using the following equation:

(1) $$\nabla \cdot \sigma \nabla \varphi =-\nabla \cdot \sigma \frac{\partial \text{A}}{\partial \text{t}}$$

where σ is the electric conductivity, as a scalar piecewise constant conductivity, using the same values in Aonuma et al. (2018). The time derivative of the magnetic vector potential $\frac{\partial \text{A}}{\partial \text{t}}$ was determined from the model using the thin-wire approximation and the Biot–Savart law. The coil consists of two wings with eleven equally spaced concentric current loops mimicking the coil in the experiment. The inner and outermost loops of the coil were 1.75 and 3.8 cm in diameter, respectively. Second, the induced electric field was calculated as follows:

(2) $$E=-\nabla \phi -\frac{\partial }{\partial \text{t}}\text{A}$$

The computed induced EF and scalar electric potential corresponded to temporal peak values at the coil operating frequency of 3 kHz (Nieminen et al., 2015). Eq. (1) was solved numerically by the finite-element method with first-order cubical elements (0.488-mm voxel of the volume conductor model), using an in-house software, as described in Laakso and Hirata (2012), which has been applied in different TMS studies (Gomez-Tames et al., 2020a; Laakso et al., 2018; Gomez-Tames et al., 2020b).

We used an anatomical volume conductor model for each participant to compute the individualized induced electric fields. The coil position and orientation and stimulation intensity in the simulation were the same as those in the experiments. Coil orientation and position were recorded using a navigation system.

### Nerve Model

The effects of the computed extracellular electric scalar field on nerve model axons placed over the Broca’s area were investigated. The smallest stimulator output intensity was obtained to elicit an action potential in a conductance-based nerve model using the following equation:

(3) $$c_{m}\frac{dV_{m,n}}{dt}=-I_{ion}+\frac{\Delta^{2}\varphi }{R}+\frac{\Delta^{2}V_{m,n}}{R}$$

where *c*_*m*_ is the membrane capacitance, *V*_*m,n*_ is the membrane potential at position *n* along the axon, and the variable *R* denotes the intra-axonal resistance between the centers of two adjacent compartments. The list of values of the nerve parameters can be found in Gomez-Tames et al. (2019). The spatial structure of the myelinated neuron consists of internodes (segments ensheathed by myelin) concatenated with nodes of Ranvier (ionic channels). The ionic current is passive in the internodes and nonlinear in the nodes, as described by the Chiu–Ritchie–Rogart–Stagg–Sweeney model (Sweeney et al., 1987).

The right-hand side of Eq. 3 corresponds to Δ^2^ϕ = ϕ(n−1)−2ϕ(n) + ϕ(n + 1), which describes the driving term of the activation. To incorporate the total induced electric field ***E*** of Eq. 2 in Eq. 3, a local linear integral of the electric field along the trajectory of the cable model is used to obtain the extracellular driving potential ϕ :

(4) ϕ=-∫E⋅dl

The terminal condition used for the proximal axon node at the seed point was clamped at 0 V.

## Results

### Experimental Pain and Perception Thresholds

**FIGURE 3:**
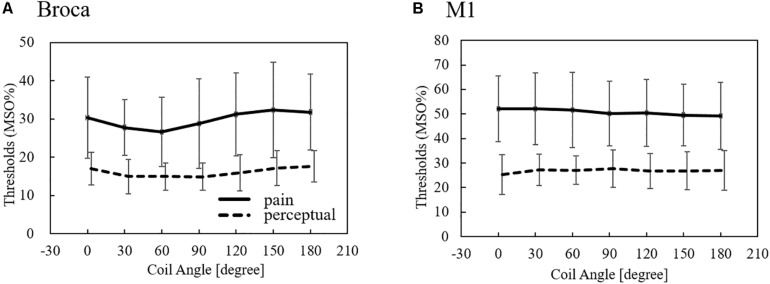
Measured perception and pain thresholds over Broca’s and M1 areas (average ± SD, *n* = 12). The thresholds are given by the MSO% of the device. SD: standard deviation.

In Figure 3, the measured average pain and perceptual thresholds are shown for different coil orientations. The threshold is given by the percentage of the maximum stimulation output of the device (MSO%). One-way analysis of variance (ANOVA) with repeated measures revealed a significant main effect of coil angle for the Broca’s area (perception, *F*_6, 66_ = 3.58; *p* < 0.01; pain, *F*_6, 66_ = 4.38; *p* < 0.001), but not for the M1 region (perception, *F*_6, 66_ = 1.21; *p* = 0.31; pain, *F*_6, 66_ = 1.40; *p* = 0.26). We found that pain and perceptual thresholds were significantly lower in the 60° than the 180° conditions (Bonferonni post-hoc tests; *p* < 0.05). No significant differences were not observed between the other conditions. These results indicate a coil angle dependency only for TMS targeting the Broca’s area. The pain/perception thresholds were not the same for the target areas. In addition, the pain threshold (%MSO) was approximately twice that of perception.

### Dosimetry of Pain and Perception Thresholds

**FIGURE 4:**
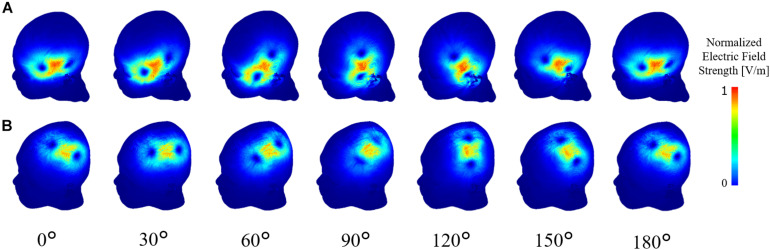
Computational dosimetry using the experimental conditions on individualized head models. Induced electric field is shown in one representative subject for TMS stimulation over the **(A)** Broca’s and **(B)** M1 areas.

Computational electromagnetic dosimetry was conducted to determine the induced electric field using the same stimulation conditions in the experiment (MSO, coil position/orientation, and individual head model). Figure 4 illustrates the induced electric field in the skin for different coil positions over the Broca’s and M1 areas.

**FIGURE 5:**
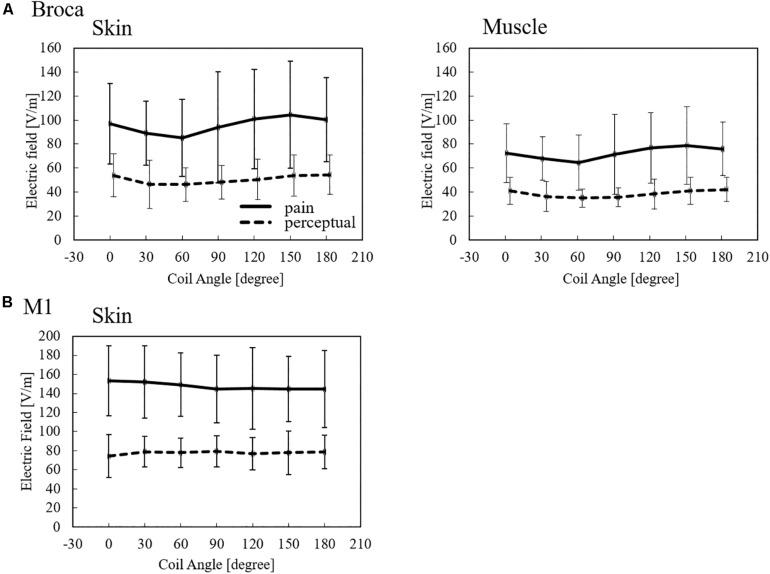
Induced electric field thresholds (average ± SD, *n* = 12) derived from measured values in non-brain tissues for **(A)** Broca’s and **(B)** M1 targets.

To investigate the consistency of the coil dependency of the measured thresholds, the internal electric field in the skin and muscle was computed using the same experimental conditions (measured MSO% threshold, coil orientation/position, and individualized head models) for each subject. As shown in Figure 5, the dependency on the coil orientation of the induced electric showed similar trends in the Broca’s and M1 areas to the measured thresholds (Figure 3).

The induced electric field strength corresponding to the pain threshold varies between 85 and 104 V/m in skin tissue and 64 and 79 V/m in the muscle for stimulation over the Broca’s area. In addition, the perception threshold varies between 46 and 53 V/m in the skin and 35 and 42 V/m in the muscle for stimulation over the Broca’s area. In the case of M1, the induced electric field thresholds were higher than those of the Broca’s area. The variation of perception thresholds between Broca’s area and M1 indicates that different perception/pain fibers are involved.

### Nerve Stimulation in Broca’s Area

**FIGURE 6:**
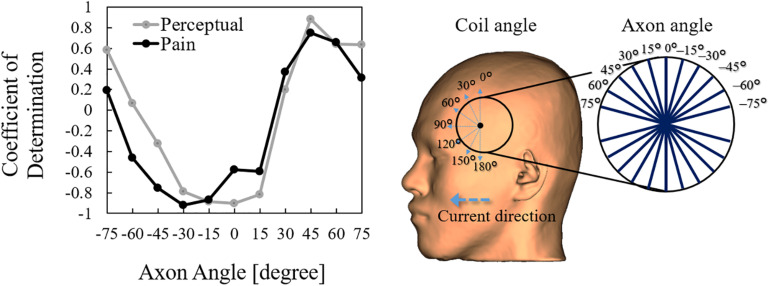
Correlation between the activation threshold (nerve) and induced electric field (computational dosimetry of experimental condition) at different coil orientations. The correlation is obtained using different axon angles above the Broca’s area.

We also derived the stimulation thresholds directly from multiscale modeling by incorporating a nerve model in the muscle over the Broca’s area (Supplementary Table 1). We then investigated the most effective orientation of the fiber below the Broca’s area and analyzed the coefficient of determination between induced electric field thresholds based on a nerve stimulation model and computational dosimetry based on the experimental measurements. We identified one effective orientation of the nerve model (45°) in which the coefficient of determination reaches the maximum value, as shown in Figure 6. For this orientation, the axon threshold lies between 31 and 79 V/m, which agrees with the dosimetry analysis of perception thresholds in the muscle (Figure 5). This indicates that pain thresholds may be derived from not only the skin tissue but also deeper tissues in Broca’s area where muscle tissue exists.

## Discussion

There is a large body of evidence that motor and speech representations in the primary motor cortex and Broca’s area have an optimal coil rotation angle (i.e., the direction of the induced current), which depends on individual neuroanatomy (Guggisberg et al., 2001; Bashir et al., 2013; Raffin et al., 2015; Sollmann et al., 2015; Stephani et al., 2016). Pain thresholds for both Broca’s area and M1 are significant differently (Tani et al., 2020). Moreover, the pain threshold shows a significant difference between coil orientations in the Broca’s area in contrast to M1 (Tani et al., 2020). This study conducted a dosimetry analysis of the induced electric field in non-brain tissues related to perception and pain thresholds for the first time to quantify the internal values and clarified differences observed between Broca’s area and M1. The computation of the induced electric field is based on the same conditions of TMS experiments to detect perception/pain thresholds when targeting the Broca’s and M1 areas, including the head model considering subject anatomy. In addition, multiscale analysis was performed to investigate the activation thresholds of neural fibers in non-brain tissue.

We confirmed that the induced electric field thresholds for pain and perception depend on the coil orientation for Broca’s area stimulation, but not for M1 stimulation. Moreover, Broca’s area stimulation has smaller induced electric field thresholds than M1 stimulation. Both observations suggest that different perception/pain fibers are involved during stimulation of the Broca’s and M1 areas. First, the non-dependence of coil orientation during M1 stimulation could be related to the stimulation of small fibers (e.g., Aδ- and C-fibers) that are approximately normal to the skin surface. Second, smaller thresholds when targeting the Broca’s area could result because thicker fibers underneath the skin are stimulated with lower thresholds following the inverse relationship between fiber thickness and stimulation threshold in deeper tissues (e.g., muscle fibers). For the first argument, the induced electric fields on the skin during M1 stimulation present similar values to Aδ-fiber stimulation thresholds for perception in the range of 65 to 130 V/m, as found in Tanaka et al. (2020). For the second argument, we characterized peripheral stimulation based on multiscale computation by modeling fibers on the muscle tissue that suggest fiber orientation similar to muscle found under the Broca’s area (e.g., temporoparietal muscle fibers). These observations indicate that pain thresholds may be derived from not only the skin tissue, but also deeper tissues, such as the muscle tissue when the Broca’s area is targeted. This was not the case for M1 due to the lack of muscle tissue, and because the perception/threshold is mostly driven by small fibers on the skin. The participants identified the pain location on the scalp as near below the center of the coil during Broca’areas stimulation, which discards pain originated from other areas, such as the eyes (K. Tani, personal communication, 2021).

Finally, the exposure level of the internal electric field in non-brain tissues was quantified for the first time in perception and pain conditions. The minimum thresholds were 40 V/m in the Broca’s area and 80 V/m in the M1 area. These values are useful when considering the perception and pain levels in standardization and medical applications. Studies aiming to achieve considerable induced electric field values in deep brain regions, such as deep TMS, need to consider the high induced fields in non-brain tissues. It has been shown that the electric field level in deep brain regions corresponds to a maximum of 50% of cortical values and 25% of scalp values (Gomez-Tames et al., 2020a). This large difference indicates the importance of considering the side effects of stimulation and the importance of dosimetry in non-brain tissues. Factors affecting the perception/pain thresholds is the TMS coil design, stimulation waveform, pain assessment for evaluation of multiple levels, and interindividual differences. For the coil design, the quantification of internal electric field thresholds reduces its effects. As future work, we will expand on dosimetry for brain tissues.

## Data Availability Statement

The raw data supporting the conclusions of this article will be made available by the authors, without undue reservation.

## Ethics Statement

The studies involving human participants were reviewed and approved by Hamamatsu University School of Medicine. The patients/participants provided their written informed consent to participate in this study.

## Author Contributions

AH and ST conceived and designed the study. ST and KT conducted the experiments. JG-T and KH conducted the simulation experiments. JG-T, KT, and KH processed the data. All authors analyzed the data, wrote the manuscript, and read and approved the manuscript.

## Conflict of Interest

The authors declare that the research was conducted in the absence of any commercial or financial relationships that could be construed as a potential conflict of interest.
