# Supplementary material for: Dosimetry Analysis in Non-brain Tissues During TMS Exposure of Broca’s and M1 Areas
Source: Front Neurosci. 2021 Feb 19;15:644951. doi: 10.3389/fnins.2021.644951 (PMC7933205; doi:10.3389/fnins.2021.644951)
Supplement: Supplementary file 1 [file Table_1.DOCX]

Supplementary Material

**Supplementary Table 1.** Induced electric fields to activate axon during TMS over the Broca’s area. Induced electric fields derived from the experimental measurement are also presented.

| Coil Angle [°] | Average Electric Field Threshold [V/m] (Standard Deviation) | | | | | | | | | | | | |
| --- | --- | --- | --- | --- | --- | --- | --- | --- | --- | --- | --- | --- | --- |
|  | Dosimetry analysis | | Axon Angle [°] | | | | | | | | | | |
|  | Percep | Pain | -75 | -60 | -45 | -30 | -15 | 0 | 15 | 30 | 45 | 60 | 75 |
| 0 | 41.2 | 72.4 | 40.3  (16.1) | 41.3  (21.1) | 62.1  (41.4) | 40.3  (21.1) | 28.2  (10.5) | 27.3  (7.2) | 33.8  (16.7) | 51.0  (30.5) | 79.0  (55.7) | 44.7  (18.6) | 50.9  (28.4) |
| 30 | 36.8 | 67.2 | 44.6  (16.9) | 47.7  (25.2) | 45.3  (17.1) | 43.3  (25.0) | 55.9  (37.6) | 39.4  (14.3) | 32.3  (12.0) | 31.9  (12.3) | 35.8  (14.6) | 43.3  (25.4) | 62.1  (33.3) |
| 60 | 36.1 | 64.4 | 51.0  (18.1) | 56.7  (37.4) | 63.2  (42.7) | 48.7  (24.1) | 53.6  (42.5) | 58.5  (27.6) | 55.0  (30.7) | 41.2  (26.3) | 31.3  (13.7) | 27.3  (9.7) | 39.9  (20.8) |
| 90 | 36.4 | 70.9 | 26.6  (5.2) | 35.5  (10.7) | 48.0  (12.0) | 43.0  (19.2) | 35.7  (13.4) | 63.5  (36.0) | 50.0  (27.0) | 45.5  (25.4) | 41.5  (18.6) | 40.7  (28.5) | 29.1  (15.5) |
| 120 | 38.6 | 75.4 | 24.5  (6.0) | 21.3  (4.1) | 26.7  (15.4) | 28.9  (19.7) | 39.6  (43.8) | 54.4  (32.9) | 40.2  (17.1) | 45.6  (17.7) | 52.5  (25.9) | 36.3  (18.0) | 47.4  (33.0) |
| 150 | 41.3 | 77.8 | 59.8  (42.8) | 34.4  (13.0) | 23.0  (7.0) | 18.1  (7.9) | 15.7  (5.3) | 20.5  (6.5) | 33.6  (25.6) | 47.3  (25.6) | 68.8  (47.6) | 50.4  (30.1) | 53.2  (24.0) |
| 180 | 42.6 | 75.9 | 72.8  (57.6) | 59.3  (32.2) | 40.4  (21.4) | 20.9  (8.6) | 14.8  (3.8) | 14.5  (3.2) | 18.3  (9.2) | 35.5  (39.8) | 60.5  (53.7) | 43.3  (25.8) | 68.3  (43.5) |
